# Supplementary material for: Characteristic Assessment of Angiographies at Different Depths with AS-OCTA: Implication for Functions of Post-Trabeculectomy Filtering Bleb
Source: J Clin Med. 2022 Mar 16;11(6):1661. doi: 10.3390/jcm11061661 (PMC8949979; doi:10.3390/jcm11061661)
Supplement: Supplementary file 1 [file jcm-11-01661-s001.zip › Supplementary File S1.pdf]

We use two sample size calculation methods, as follows.

Methods 1 and 2 were calculated by gpower sample size calculation software
The sample size was expanded according to the 20% loss of follow-up rate.

Method 1

t tests - Means: Difference between two independent means (two groups)

**Analysis:**	A priori: Compute required sample size		
**Input:**	Tail(s)	=	Two
	Effect size d	=	1.5339300
	α err prob	=	0.05
	Power (1-β err prob)	=	0.9
	Allocation ratio N2/N1	=	2
**Output:**	Noncentrality parameter δ	=	3.5424596
	Critical t	=	2.0738731
	Df	=	22
	Sample size group 1	=	8
	Sample size group 2	=	16
	Total sample size	=	24

Actual power = 0.9227879

Final smaple size = 24*120% = 28.8≈29

Method 2

F tests - Linear multiple regression: Fixed model, R^2 deviation from zero

**Analysis:**	A priori: Compute required sample size		
**Input:**	Effect size f^2	=	0.48
	α err prob	=	0.05
	Power (1-β err prob)	=	0.9
	Number of predictors	=	3
**Output:**	Noncentrality parameter λ	=	16.3200000
	Critical F	=	2.9222772
	Numerator df	=	3
	Denominator df	=	30
	Total sample size	=	34
	Actual power	=	0.9029060

Final smaple size = 34 * 120% = 40.8≈41
