# Supplementary material for: Characteristic Assessment of Angiographies at Different Depths with AS-OCTA: Implication for Functions of Post-Trabeculectomy Filtering Bleb
Source: J Clin Med. 2022 Mar 16;11(6):1661. doi: 10.3390/jcm11061661 (PMC8949979; doi:10.3390/jcm11061661)
Supplement: Supplementary file 1 [file jcm-11-01661-s001.zip › Supplementary Table S1.pdf]

Supplementary Table S1. Argument assignment table

Arguments	Value Assignment
IBAGS	Taking category 0 as a reference
	Dummy variable X1: "category 1"=1; "category 0,2,3"= 0
	Dummy variable X2: "category 2"=1; "category 0,1,3"= 0
	Dummy variable X3: "category 3"=1; "category 0,1,2"= 0
KGS	Taking "Type 1" as a reference
	Dummy variable X1: "Type 2"=1; "Type 1,3,4"= 0
	Dummy variable X2: "Type 3"=1; "Type 1,2,4"= 0
	Dummy variable X3: "Type 4"=1; "Type 1,2,3"= 0

IBAGS= Indiana Bleb Grading Appearance Scale; KGS= Kenfeld grading system.
