# Supplementary material for: Characteristic Assessment of Angiographies at Different Depths with AS-OCTA: Implication for Functions of Post-Trabeculectomy Filtering Bleb
Source: J Clin Med. 2022 Mar 16;11(6):1661. doi: 10.3390/jcm11061661 (PMC8949979; doi:10.3390/jcm11061661)
Supplement: Supplementary file 1 [file jcm-11-01661-s001.zip › Supplementary Table S2.pdf]

Supplementary Table S2. Detailed Baseline Characteristics of Study Subjects

	Follow-up time (year)	Preoperative IOP (mmHg)	Postoperative IOP (mmHg)	The percentage of IOP reduction (%)	Preoperative topical ocular hypotensive drugs (n)	Preoperative use of PGs (n)	Postoperative topical ocular hypotensive drugs (n)	Postoperative use of PGs (n)
1	1.5	14	7	47	2	0	0	0
2	0.5	14	7	49	0	0	0	0
3	5.0	24	18	25	3	1	0	0
4	1.0	25	13	47	0	0	1	1
5	3.0	22	14	35	3	1	0	0
6	11.0	36	17	53	3	1	1	0
7	10.0	41	14	65	3	1	1	1
8	10.0	20	15	27	3	1	1	1
9	6.0	21	5	77	3	1	0	0
10	1.0	19	13	30	3	1	0	0
11	10.0	17	12	28	3	1	0	0
12	2.0	17	6	63	0	0	0	0
13	0.5	13	7	43	1	0	0	0
14	10.0	22	16	29	0	0	2	1
15	2.0	26	18	32	0	0	0	0
16	2.0	36	19	48	0	0	0	0
17	1.0	24	17	28	3	1	0	0
18	1.0	21	11	46	3	1	0	0
19	1.5	35	14	60	0	0	0	0
20	2.5	17	11	32	0	0	2	0
21	5.0	40	16	60	0	0	0	0
22	1.5	38	10	74	0	0	0	0
23	20.0	48	19	60	3	0	3	1
24	20.0	25	19	23	0	0	3	1
25	1.0	28	19	32	0	0	0	0
26	14.0	20	9	53	3	1	3	1
27	2.0	29	12	59	3	1	0	0
28	0.5	26	14	46	3	1	0	0
29	0.5	23	39	-70	0	0	1	0
30	1.0	32	38	-19	3	1	3	1
31	1.0	44	36	18	2	0	3	1
32	1.0	41	28	31	0	0	1	0
33	0.5	19	27	-42	3	1	1	0
34	0.5	16	24	-51	3	1	0	0
35	5.0	19	23	-21	0	0	0	0
36	1.0	45	43	5	1	0	0	0

37	0.5	40	25	37	3	0	0	0
38	12.0	19	28	-46	1	0	3	1
39	12.0	26	23	10	2	0	3	1
40	2.0	25	23	6	0	0	0	0
41	0.5	22	22	2	3	1	0	0
42	2.0	23	26	-13	0	0	0	0
43	2.0	24	30	-25	0	0	0	0
44	5.0	35	24	31	3	1	3	1
45	0.5	28	22	21	0	0	0	0
46	1.0	28	26	7	1	0	3	1

IOP = intraocular pressure, PG= prostaglandin.
