# Supplementary material for: Characteristic Assessment of Angiographies at Different Depths with AS-OCTA: Implication for Functions of Post-Trabeculectomy Filtering Bleb
Source: J Clin Med. 2022 Mar 16;11(6):1661. doi: 10.3390/jcm11061661 (PMC8949979; doi:10.3390/jcm11061661)
Supplement: Supplementary file 1 [file jcm-11-01661-s001.zip › Supplementary Table S3.pdf]

Supplementary Table S3. Univariate and Multivariate Linear Regression Analysis for IOP

Variable		Univariable Model		Multivariable Model 1*		Multivariable Model 2+	
		Coefficient (95% CI)	*p* Value	Coefficient (95% CI)	*p* Value	Coefficient (95% CI)	*p* Value
SL							
	VD (%)	0.515 (-0.336-1.365)	0.229				
	VDI (pixel-1)	**1.143 (-0.085-2.201)**	**0.035**			n/a	0.15
TL							
	VD (%)	**0.648 (0.472-0.823)**	**0.000**	**0.648 (0.472-0.823)**	**0.000**		
	VDI (pixel-1)	**1.659 (1.250-2.069)**	**<0.001**			**1.659 (1.250-2.069)**	**0.000**
DL							
	VD (%)	**0.599 (0.431-0.767)**	**0.000**	n/a	0.677		
	VDI (pixel-1)	**1.526 (1.109-1.943)**	**0.000**			n/a	0.183

IOP= intraocular pressure; SL = superficial layer; TL = Tenon's layer; DL = deep layer; CI= confidence interval; VD= Vessel density; VDI= Vessel diameter index; n/a= not applicable.

P values are shown in bold as statistically significant.

All variables with $p < 0.1$ in a univariable regression analysis was selected for multivariable regression analysis.

* Stepwise regression for VD in TL and DL.

+ Stepwise regression for VDI in SL, TL, and DL.

# Not included in multivariate model after stepwise regression.
