# Supplementary material for: Characteristic Assessment of Angiographies at Different Depths with AS-OCTA: Implication for Functions of Post-Trabeculectomy Filtering Bleb
Source: J Clin Med. 2022 Mar 16;11(6):1661. doi: 10.3390/jcm11061661 (PMC8949979; doi:10.3390/jcm11061661)
Supplement: Supplementary file 1 [file jcm-11-01661-s001.zip › Supplementary Table S4.pdf]

Supplementary Table S4. Univariate and Multivariate Logistic Regression Analysis for Surgical Outcome

Variable	Univariable Model		Multivariable Model 1*		Multivariable Model 2+	
	OR (95% CI)	*P* Value	OR (95% CI)	*P* Value	OR (95% CI)	*P* Value
Conjunctival layer						
VD (%)	1.192 (0.972-1.460)	0.091	n/a#	0.175		
VDI (pixel-1)	1.312 (0.970-1.774)	0.078			n/a	0.595
Tenon's layer						
VD (%)	**1.448 (1.165-1.799)**	**0.001**	**1.448 (1.165-1.799)**	**0.001**		
VDI (pixel-1)	**1.862 (1.370-2.530)**	**0.000**			**1.862 (1.370-2.530)**	**0.000**
Scleral layer						
VD (%)	**1.308 (1.128-1.517)**	**0.000**	n/a#	0.376		
VDI (pixel-1)	**1.724 (1.315-2.260)**	**0.000**			n/a	0.105

OR= odds ratio; CI= confidence interval; VD= Vessel density; VDI= Vessel diameter index; n/a= not applicable; n/a= not applicable.

P values are shown in bold as statistically significant.

All variables with $p < 0.1$ in a univariable regression analysis was selected for multivariable regression analysis.

* Stepwise regression for conjunctival, Tenon's and scleral VD.

\+ Stepwise regression for conjunctival, Tenon's and scleral VDI.

\# Not included in multivariate model after stepwise regression.
